# Acute effects of blood flow restriction training at various arterial occlusion pressures on muscle activation, blood lactate responses, and RPE in healthy adult males

**DOI:** 10.3389/fphys.2025.1620294

**Published:** 2025-09-10

**Authors:** Hao Zhu, Zhaowen Tan, Nianyun Zhang, Yang Li, Hu Qi

**Affiliations:** ^1^ Department of Physical Education, Nanjing Xiaozhuang University, Nanjing, China; ^2^ Nanjing Sport Institute, Nanjing, China; ^3^ School of Physical Education, Anshan Normal University, Anshan, China

**Keywords:** blood flow restriction training, arterial occlusion pressure, muscle activity, lactate, ratings of perceived exertion, repeated-measures ANOVA, partial eta-squared

## Abstract

**Background:**

Blood flow restriction training (BFRT) can induce significant muscle activation and metabolic stress at low loads. However, the acute physiological and perceptual responses to different arterial occlusion pressures (AOPs) remain unclear. This study aimed to examine the effects of varying AOP levels on muscle activation, blood lactate concentration, and ratings of perceived exertion (RPE) during low-load resistance exercise in healthy young males.

**Methods:**

Sixteen healthy males (20.4 ± 1.5 years) participated in a single-group, repeated-measures study. Each performed barbell back squats (20% 1RM) under four AOP conditions: 0%, 60%, 70%, and 80% AOP. Muscle activation (%MVC), blood lactate concentrations, and RPE were assessed. One-way and two-way repeated-measures ANOVAs were used to analyze the outcomes across pressure and time conditions.

**Results:**

Muscle activation increased significantly at 70% and 80% AOP compared to 0% and 60% (e.g., semitendinosus: F (3, 45) = 15.79, p < 0.001, ηp^2^ = 0.51, 95% CI [0.14, 0.40]), with no difference between 70% and 80% AOP. Blood lactate concentrations increased significantly post-exercise under 70% and 80% AOP (F (3, 45) = 4.82, p = 0.005, ηp^2^ = 0.24, 95% CI [0.03, 0.22]), although the main effect of pressure was not significant across time points (F (3, 45) = 1.63, p = 0.192, ηp^2^ = 0.08, 95% CI [0.01, 0.13]). RPE increased progressively with pressure (F (1.80, 26.94) = 25.34, p < 0.001, ηp^2^ = 0.63, 95% CI [0.28, 0.66]), and was highest at 80% AOP.

**Conclusion:**

70%–80% AOPs elicited greater acute neuromuscular and metabolic responses compared to lower pressures, with 70% AOP achieving similar physiological outcomes as 80% but with lower perceived exertion. These findings provide practical guidance for selecting relative occlusion pressures during BFRT. Further studies are warranted to explore long-term training adaptations at these pressures.

## 1 Introduction

Few studies have directly compared the acute neuromuscular and perceptual responses to Blood flow restriction training (BFRT) at 60%, 70%, and 80% of arterial occlusion pressure (AOP) in healthy adults. This gap limits our understanding of how different AOP levels modulate training responses and tolerability. BFRT is a brief, low-intensity resistance training method that employs specialized compression devices (inflatable cuffs) applied to proximal limbs to partially occlude blood flow. This technique induces a hypoxic environment that enhances metabolic stress within targeted muscles, thereby promoting hypertrophy (Jessee et al., 2018; Pignanelli et al., 2021). Known as “KAATSU” in Japan, BFRT has gained traction across athletic performance enhancement and clinical rehabilitation (Bond et al., 2019; Hackney et al., 2018; Geng et al., 2021). Research indicates that while utilizing substantially lower intensities (20%–40% 1RM) than traditional resistance training, BFRT achieves comparable muscle hypertrophy with reduced muscle damage (Loenneke et al., 2012). This efficacy stems from high metabolic stress under appropriate blood flow restriction (Pearson and Hussain, 2015; Yuan et al., 2023), making pressure level selection a critical determinant of outcomes alongside exercise type and intensity (Mouser et al., 2017).

Theoretically, cuff pressure during BFRT must balance venous occlusion without arterial compromise (Loenneke et al., 2014). Therefore, to achieve this balance, pressure should be individualized based on cuff placement, limb circumference, subcutaneous fat thickness, and vascular health (Spranger et al., 2015). Excessively high pressures risk discomfort, pain, and vascular complications (Nascimento et al., 2022; Tabata et al., 2016), while insufficient pressures limit metabolic stimulus (Mouser et al., 2017).

Critically, individualized pressure settings are essential for BFRT efficacy and safety, as factors such as limb circumference, cuff width, and body position significantly influence the absolute pressure required to achieve consistent vascular occlusion. For example, larger limbs or narrower cuffs necessitate higher pressures to attain equivalent occlusion levels, meaning that arbitrary fixed pressures (e.g., a fixed absolute value 220 mmHg for all participants) may cause under-occlusion in individuals with larger limbs or over-occlusion in those with smaller limbs (Cook et al., 2014; Segal et al., 2015; Yasuda et al., 2014). This methodological limitation compromises both safety (e.g., vascular injury risk) and efficacy (inconsistent metabolic stimuli) (Patterson et al., 2019). Although some studies have attempted to use percentages of systolic blood pressure to set BFRT occlusion pressure (Abe et al., 2010; Hunt et al., 2016; Yasuda et al., 2006), it is an unreliable predictor of the occlusion pressure required in BFRT due to poor correlation with anthropometric determinants like limb circumference and subcutaneous fat thickness (Hunt et al., 2016; McEwen et al., 2019; de Queiros et al., 2024). Consequently, current guidelines advocate individualized pressures based on arterial occlusion pressure (AOP) to standardize the degree of blood flow restriction across diverse populations (Jessee et al., 2016; Patterson et al., 2019). AOP is a key factor in BFRT prescription, defined as the minimum cuff pressure required to completely occlude arterial blood flow to the limb. Determining each person’s AOP allows clinicians to set cuff inflation as a percentage of this value, ensuring a comparable degree of blood flow restriction across individuals. While AOP standardization improves reliability (Hughes et al., 2017; Masri et al., 2016), optimal percentages remain debated. Recent evidence highlights this balance: occlusion pressures below 50% AOP or near full occlusion (≥80% AOP) tend to produce suboptimal strength improvements, whereas mid-range pressures (50%–80% AOP) are more effective (Das and Paton, 2022; Roehl et al., 2023). These findings underscore the need to identify an optimal occlusion pressure that balances efficacy and tolerability in BFRT.

A recent review found that over 86% of studies of BFRT included no justification of the AOP chosen in their methodology (Clarkson et al., 2020). Given the centrality of AOP to the BFR stimulus, it is important to clarify how varying relative occlusion levels acutely affect performance and perceptual outcomes. To date, few studies have directly compared BFRT at different percentages of AOP in healthy adults. Therefore, this study employed Doppler ultrasound to directly measure AOP and investigated the acute effects of low-load resistance exercise performed with BFRT at various percentages of individually measured AOP in healthy young men. By examining neuromuscular and perceptual responses at each pressure level, this work aims to inform evidence-based guidelines for selecting BFR pressures that maximize training benefits while minimizing adverse responses. The null hypothesis (H_0_) of this study is that there are no significant differences in muscle activation (%MVC), blood lactate concentration, or ratings of perceived exertion (RPE) across BFRT conditions set at 60%, 70%, and 80% AOP. The alternative hypothesis (H_1_) is that BFRT at 70% and 80% AOP will elicit significantly greater muscle activation and blood lactate accumulation, along with higher RPE scores, compared to 60% AOP and the control condition.

## 2 Research methods

### 2.1 Research participants

The required sample size was calculated using G*Power (version 3.1.9.7) for a repeated-measures ANOVA with one within-subject factor (occlusion pressure) at four levels: 0%, 60%, 70%, and 80% AOP. Parameters were set as follows: α = 0.05, statistical power (1 - β) = 0.80, number of measurements = 4, and an anticipated medium effect size of partial η^2^ = 0.06. Based on these assumptions, the analysis yielded a required minimum sample size of 16 participants. Therefore, sixteen healthy male university students (age 20.4 ± 1.5 years) were recruited to ensure adequate statistical power.

Prior to the experiment, we provided all the participants with a comprehensive explanation of the study’s purpose, procedures, and potential risks. We also distributed health questionnaires and consent forms to those who volunteered and obtained their consent to participate. This study was approved by the Institutional Review Board (IRB) of University N. Inclusion criteria were as follows: (1) males aged 18–30 years; (2) free of any known cardiovascular, metabolic, or neuromuscular diseases; (3) capable of performing low-load resistance training; (4) willing to undergo capillary blood sampling and without a history of needle phobia or vasovagal syncope. Exclusion criteria included: (1) any diagnosed cardiovascular disease, peripheral vascular disease, or history of deep vein thrombosis; (2) abnormal electrocardiogram findings at rest or after exercise; (3) musculoskeletal disorders that would interfere with training; (4) resting systolic blood pressure ≥160 mmHg or diastolic pressure ≥100 mmHg; (5) any physician-determined contraindications to exercise; (6) known psychological, cognitive, or eating disorders; (7) history of diabetes or cancer; (8) individuals requiring medically supervised physical activity; (9) > 5 mm difference in thigh circumference between legs. All participants met the inclusion criteria and completed the study protocol without adverse events.

### 2.2 Experimental design and procedure

#### 2.2.1 Pre-experiment screening

During the initial visit, the participants were required to complete a Physical Activity Readiness Questionnaire (PAR-Q) and provide written informed consent. As part of the pre-experiment screening, all participants were instructed to rest quietly for 5–10 min upon arrival at the laboratory. Subsequently, the arterial occlusion pressure (AOP) for the lower limbs was measured to determine the appropriate pressure levels for subsequent training sessions, and the one-repetition maximum (1RM) for squats was assessed to establish exercise intensity.

**FIGURE 1:**
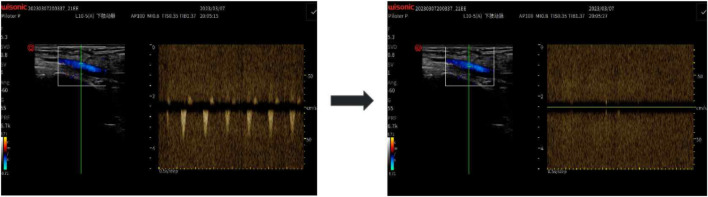
Doppler Ultrasound Measurement of Arterial Occlusion Pressure (AOP): Using a handheld Doppler probe (WISONIC Piloter S), blood flow in the posterior tibial artery was monitored while a thigh cuff was inflated. AOP was defined as the pressure at which the Doppler signal disappeared.

The AOP at the posterior tibial artery was measured in a standing position using color Doppler ultrasound (Doppler probe WISONIC Piloter S, CHN). The procedure involved placing a pressure cuff around the proximal thigh, and gradually increasing the pressure while monitoring blood flow at the posterior tibial artery with a handheld Doppler probe. The minimum pressure at which blood flow ceased was recorded as the participant’s AOP (i.e., 100% AOP). To ensure measurement consistency, all AOP assessments were conducted by a trained specialist with over 3 years of experience in vascular ultrasound. Each measurement was repeated twice, and the average value was recorded as the final AOP. In cases where the two values differed by more than 5 mmHg, a third measurement was taken, and the closest two values were averaged. The intraclass correlation coefficient (ICC) between the first and second measurements was 0.993, indicating excellent reliability. The mean AOP across all participants was 240.50 ± 22.40 mmHg. This measurement approach was adopted based on best-practice recommendations for vascular occlusion pressure assessment using ultrasound (Hart et al., 2022). Based on this measurement, the target pressure levels for training sessions were calculated as 60%, 70%, and 80% of AOP. A representative Doppler ultrasound image of AOP assessment is shown in Figure 1.

#### 2.2.2 Experimental procedure

This study employed a single-group repeated measures design. All participants (n = 16) completed four testing sessions in the same fixed order: 0% AOP (week 1), 60% AOP (week 2), 70% AOP (week 3), and 80% AOP (week 4). Although a fully counterbalanced design is generally preferred to minimize order effects, complete randomization was not feasible in this study due to practical limitations related to equipment availability, scheduling constraints, and participant adherence. As a partial mitigation strategy, we implemented a minimum 1-week washout period between sessions to reduce carryover effects (Abbuhl et al., 2013; Alwin, 2007; Brooks, 2012; Frost, 2015). Nonetheless, the absence of full counterbalancing is acknowledged as a limitation of the study design. The exercise protocol for this study is shown in Table 1.

**TABLE 1:** The exercise protocol.

Procedures	Weeks	Pressure (mmHG)	Exercise parameters	Duration (min)
Warm-up	1–4		Dynamic Stretching Exercises	10 min
Main Exercise	1	0% AOP	Exercise Type: Squats Exercise Intensity: 20% 1RM Number of Sets: 4 Sets Repetitions 30 + 15 + 15 + 15	10–15 min
	2	60% AOP		10–15 min
	3	70% AOP		10–15 min
	4	80% AOP		10–15 min
Cool-down	1–4		Slow Walking and Stretching Exercises	10 min

On each visit, participants arrived at the laboratory at a predetermined time and remained seated for 5 min. A professional nurse collected approximately 2 mL of blood to measure blood lactate concentration using the EKF Lactate Scout+ (Germany). Following a 10-min rest, participants performed a standardized barbell back-squat protocol consisting of four sets (30 + 15+15 + 15 repetitions), with a 1-min rest between each set. Each subject descended to a depth with the hips below the level of the knees (i.e., a full squat), following a controlled 2 s eccentric descent, 1 s isometric pause at the bottom, and 2 s concentric ascent for each repetition. A digital metronome (set at 60 BPM) was used to provide auditory pacing throughout the sets, ensuring consistency of repetition cadence across all participants. All squats were performed at a low relative load (20% of individual 1RM) on the barbell. This low-load is consistent with typical BFR protocols. To ensure consistency, participants received instruction and practice sessions on proper squat technique and tempo before data collection. During the squat exercise, surface electromyography (sEMG) signals of the lower limb muscles and ratings of perceived exertion (RPE; Borg 6–20 scale) were collected. RPE was recorded immediately after the completion of the final repetition in each condition, corresponding to the end of the fourth set. This approach was chosen to reflect the cumulative perceptual load of the entire session under each pressure level. Blood lactate levels were measured immediately post-exercise and again after a 15-min recovery period.

Surface electromyography (EMG) was utilized to conduct a pre-exercise RMS_MVC_ test to standardize RMS signals during squats (Konrad, 2005). Participants were instructed to abstain from caffeine and alcohol for 12 h and avoid vigorous exercise before each visit. All exercise sessions were conducted between 9:00 a.m. and 12:00 p.m. to minimize daily metabolic fluctuations. All parameters and variables were assessed in a temperature-controlled (20 °C–22 °C), quiet laboratory environment. Before the exercise session, participants completed a 10-min standard warm-up. A 50 mm inflatable pressure cuff (Kaatsu Global Inc., United States) was applied to the participants’ thighs to facilitate blood flow restriction adaptation. The pressure level for each session was determined based on the AOP measured during preliminary screening. The designated pressure was maintained during exercise, with participants closely monitored for adverse symptoms. If excessive pressure was reported, it was reduced by 10 mmHg per instance. Upon completion of all exercises, participants engaged in a 10-min stretching cool-down.

##### 2.2.2.1 Surface EMG signal measurement method

Surface electromyography (sEMG) electrodes were attached to six major muscle groups of the dominant leg to measure muscle activation: rectus femoris (RF), vastus medialis oblique (VMO), vastus lateralis (VL), biceps femoris long head (BF), semitendinosus (SM), and gluteus maximus (GM). Electrode placement followed the SENIAM (Surface ElectroMyoGraphy for the Non-Invasive Assessment of Muscles) guidelines, with bipolar Ag/AgCl electrodes aligned parallel to the muscle fibers, positioned midway between the origin and insertion points (Hermens et al., 2000). Inter-electrode distance was maintained at 20 mm. Skin at each site was shaved, abraded, and cleansed with 70% alcohol to reduce impedance.

**FIGURE 2:**
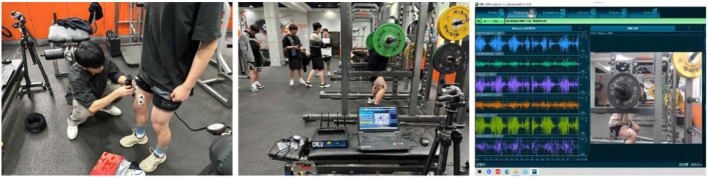
Surface Electromyography (sEMG) Setup and Recording: Surface Electromyography (sEMG) electrodes were placed on six dominant-leg muscles (RF, VL, VM, BF, SM, GM) following SENIAM guidelines. The left panel shows electrode placement preparation; the middle shows the real-time recording setup; and the right displays the raw EMG signals collected via the Ultium-EMG system (Noraxon, United States) during resistance exercise.

Surface EMG signals were collected using a wireless Ultium-EMG system (Noraxon, United States) at a sampling rate of 2,000 Hz. The raw signals were bandpass filtered using a fourth-order Butterworth filter (cutoff frequency: 20–450 Hz), then full-wave rectified and smoothed via a moving root mean square (RMS) window. The surface EMG measurements are shown in Figure 2.

To normalize EMG activity, maximal voluntary contraction (MVC) tests were performed for each muscle group prior to the exercise trials. Each MVC was held for approximately 5 s using standardized manual muscle testing (MMT) positions reflecting the primary function of the muscle. Two MVC trials were recorded for each muscle, and the RMS signal from the middle 3 s (excluding the first and last second) of each trial was averaged and used as the normalization reference. The normalized EMG activity during squat exercises was expressed as a percentage of MVC (%MVC), calculated using the formula: [%MVC = (RMS during leg muscle activity/RMS during MVC) × 100].

### 2.3 Statistical methods

The data were processed using SPSS 26.0 to calculate the Mean and Standard Deviation (M ± SD), with Standard Errors (SE) additionally reported to support interpretation in line with the research objectives. The Shapiro‒Wilk test confirmed that all the data followed a normal distribution. One-way repeated-measures ANOVA was conducted to analyze the muscle activation levels (%MVC) of the major lower limb muscles and RPE Scores under different pressure conditions. Two-way repeated-measures ANOVA was conducted to analyze the changes in blood lactate concentration over time under different pressure conditions. Mauchly’s test of sphericity was performed for all repeated-measures ANOVAs. When the assumption of sphericity was violated (Mauchly’s W p < 0.05), Greenhouse-Geisser correction was applied to adjust the degrees of freedom and control Type I error inflation. This adjustment is widely accepted in psychophysiological studies involving perceptual outcomes such as RPE (Vasey and Thayer, 1987; Greenhouse and Geisser, 1959). Post hoc tests were performed using the Bonferroni correction to adjust for multiple comparisons and control the family-wise error rate. Statistical significance was set at p < 0.05.

## 3 Results

### 3.1 Comparison of %MVC muscle activation levels under different pressure conditions

Table 2 presents the means ± SD with SE of normalized EMG activation (%MVC) for six major lower limb muscles—rectus femoris (RF), vastus lateralis (VL), vastus medialis (VM), biceps femoris (BF), semitendinosus (SM), and gluteus maximus (GM)—across four different AOP conditions (0%, 60%, 70%, 80%), with a final sample size of n = 16.

**TABLE 2:** M ± SD (SE) of %MVC for lower limb muscles at different pressure levels (n = 16).

Pressure	0%AOP	60%AOP	70%AOP	80%AOP
Muscle				
Rectus Femoris (RF)	44.54 ± 9.36 (SE = 2.34)	49.04 ± 7.87 (SE = 1.97)	55.12 ± 10.07*† (SE = 2.52)	53.65 ± 7.47*† (SE = 1.87)
Vastus Lateralis (VL)	45.20 ± 8.99 (SE = 2.25)	45.79 ± 7.14 (SE = 1.79)	52.80 ± 7.38*† (SE = 1.84)	51.30 ± 6.36*† (SE = 1.59)
Vastus Medialis (VM)	45.75 ± 9.58 (SE = 2.40)	48.87 ± 8.36 (SE = 2.09)	58.55 ± 12.2*† (SE = 3.05)	58.20 ± 10.43*† (SE = 2.61)
Biceps Femoris (BF)	39.99 ± 8.56 (SE = 2.14)	40.67 ± 5.9 (SE = 1.47)	53.75 ± 11.87*† (SE = 2.97)	49.67 ± 10.66*† (SE = 2.66)
Semitendinosus (SM)	39.68 ± 10.5 (SE = 2.62)	40.61 ± 8.51 (SE = 2.13)	53.66 ± 8.23*† (SE = 2.06)	51.47 ± 11.26*† (SE = 2.82)
Gluteus Maximus (GM)	42.03 ± 9.76 (SE = 2.44)	42.31 ± 7.67 (SE = 1.92)	53.53 ± 11.35*† (SE = 2.84)	49.85 ± 9.57*† (SE = 2.39)

Note. %MVC, percentage of maximal voluntary contraction; AOP, arterial occlusion pressure; *p < 0.05 vs. 0% AOP; † p < 0.05 vs. 60% AOP.

Table 3 summarizes the results of repeated-measures ANOVA for each muscle group, reporting F-values, degrees of freedom, p-values, partial η^2^, and 95% confidence intervals. There was a significant main effect of pressure condition on muscle activation across all muscles analyzed. For example, rectus femoris activation differed significantly across conditions, F (3, 45) = 8.66, p < 0.001, ηp^2^ = 0.37, 95% CI [0.07, 0.29]. Similarly, for the semitendinosus, F (3, 45) = 15.79, p < 0.001, ηp^2^ = 0.51, 95% CI [0.14, 0.40].

**TABLE 3:** Results of repeated measures ANOVA for %MVC at different pressure levels.

Muscle	F (df_1_, df_2_)	p-value	Partial η^2^	95% CI for partial η^2^
Rectus Femoris (RF)	8.66 (3, 45)	<0.001	0.37	[0.07, 0.29]
Vastus Lateralis (VL)	6.41 (3, 45)	0.00104	0.30	[0.05, 0.25]
Vastus Medialis (VM)	13.93 (3, 45)	<0.001	0.48	[0.12, 0.38]
Biceps Femoris (BF)	12.63 (3, 45)	<0.001	0.46	[0.11, 0.36]
Semitendinosus \(SM)	15.79 (3, 45)	<0.001	0.51	[0.14, 0.40]
Gluteus Maximus (GM)	6.19 (3, 45)	0.00131	0.29	[0.04, 0.25]

Note. %MVC, percentage of maximal voluntary contraction; AOP, arterial occlusion pressure.

**FIGURE 3:**
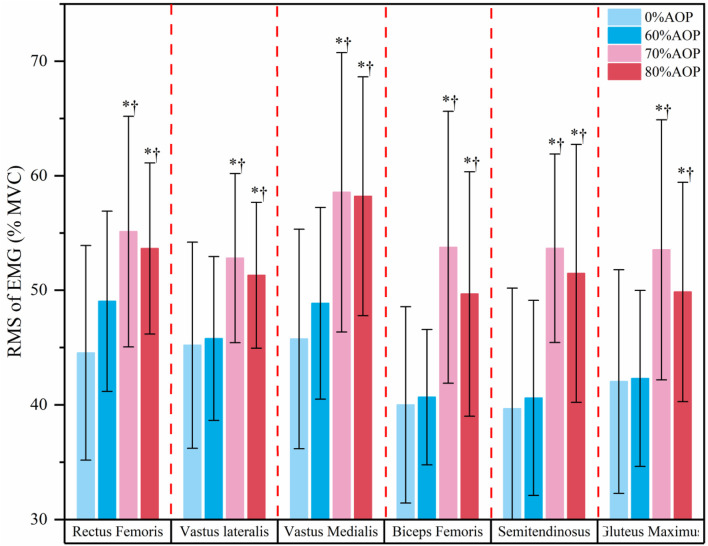
Post-hoc Test Results for the %MVC of the Major Lower Limb Muscles Under Different Pressure Levels. $\left(*p\;<\;0.05\text{ vs}.\;0\%\text{ AOP};\;†p\;<\;0.05\text{ vs}.\;60\%\text{ AOP}\right)$ .

Figure 3 displays the post hoc test results for the %MVC of the six major lower limb muscles under different AOP levels. Specifically, muscle activation at 70% and 80% AOP was significantly greater compared to 0% and 60% AOP (p < 0.05). These results indicate that higher occlusion pressures elicited greater muscle recruitment during the low-load exercise. Notably, muscle activation at 70% AOP did not significantly differ from that at 80% AOP (p > 0.05), indicating a plateau in muscle recruitment beyond 70% AOP. These results suggest that during low-load squat exercises, pressures exceeding 70% AOP may not confer additional neuromuscular activation benefits.

### 3.2 Comparison of blood lactate concentration changes and RPE scores under different pressure conditions

Table 4 summarizes the results of a two-way repeated-measures ANOVA assessing the effects of occlusion pressure and time on blood lactate concentrations. There was a significant main effect of time, F (2, 30) = 151.56, p < 0.001, ηp^2^ = 0.72, 95% CI [0.74, 0.90], indicating that lactate levels changed significantly over time. A significant time × pressure interaction was also observed, F (6, 90) = 4.92, p < 0.001, ηp^2^ = 0.20, 95% CI [0.03, 0.10], suggesting that changes in lactate levels over time differed across pressure conditions. However, the main effect of pressure was non-significant, F (3, 45) = 1.63, p = 0.192, ηp^2^ = 0.08, 95% CI [0.01, 0.13], indicating that pressure level alone did not significantly affect lactate levels. These findings suggest that blood lactate accumulation is primarily time-dependent but is significantly modulated by the interaction between time and pressure level.

**TABLE 4:** Difference analysis of blood lactate concentration at different pressure levels and time points.

Source	F (df_1_, df_2_)	p-value	Partial η^2^	95% CI for partial η^2^
time	151.56 (2, 30)	<0.001	0.72	[0.74, 0.90]
pressure	1.63 (3, 45)	0.192	0.08	[0.01, 0.13]
time × pressure	4.92 (6, 90)	<0.001	0.20	[0.03, 0.10]

Table 5 presents descriptive statistics and simple main effects analyses of blood lactate at each time point across four pressure conditions (n = 16). All values are reported as mean ± SD (SE). Post-exercise lactate concentrations (at 0 and 15 min) were significantly higher at 70% and 80% AOP compared to 0% AOP, indicating increased metabolic stress at higher occlusion levels. Notably, 70% and 80% AOP produced statistically similar lactate responses, suggesting a possible saturation point in metabolic stress induction. No significant group differences were found at baseline (Pre-ex), but significant differences emerged immediately post-exercise (0 min), F (3, 45) = 4.82, p = 0.005, ηp^2^ = 0.194. These differences diminished at 15 min post-exercise, F (3, 45) = 2.05, p = 0.116, ηp^2^ = 0.093.

**TABLE 5:** M ± SD (SE) of blood lactate concentration (mmol.L^-1^) and simple effects test results at different pressure levels and time points (n = 16).

Group	Pre-ex	Post-ex 0 min	Post-ex 15 min	F	P-value	Post-Hoc Test
Time						
0% AOP	4.13 ± 2.60 (SE = 0.65)	11.51 ± 5.56 (SE = 1.39)	6.80 ± 4.14 (SE = 1.04)	17.21	<0.001	2 > 3 >1
60% AOP	3.28 ± 3.28 (SE = 0.69)	12.63 ± 4.50 (SE = 1.12)	10.74 ± 5.55 (SE = 1.39)	32.45	<0.001	2, 3 > 1
70% AOP	3.91 ± 3.91 (SE = 0.67)	16.98 ± 6.11 (SE = 1.53)	8.49 ± 4.47 (SE = 1.12)	53.97	<0.001	2 > 3 > 1
80% AOP	3.64 ± 3.64 (SE = 0.58)	16.45 ± 3.24 (SE = 0.81)	7.79 ± 4.39 (SE = 1.10)	52.29	<0.001	2 > 3> 1
F	0.32	4.82	2.05			
P-value	0.811	0.005	0.116			
Post-Hoc Test		c,d > b,a				

Note. AOP, arterial occlusion pressure; 1: Pre-ex; 2: Post-ex 0 min; 3: Post-ex 15 min.

a, 0% AOP; b, 60% AOP; c, 70% AOP; d, 80% AOP.

Table 6 presents the means ± SD with SE of Rating of Perceived Exertion (RPE) scores across four occlusion pressure levels (n = 16). RPE values increased progressively with rising occlusion pressure, from 12.44 ± 2.87 (SE = 0.72) at 0% AOP to 16.38 ± 1.15 (SE = 0.29) at 80% AOP.

**TABLE 6:** M ± SD (SE) of RPE at different pressure levels (n = 16).

Group	0%AOP	60%AOP	70%AOP	80%AOP
Pressure				
RPE Scores (Borg Scale)	12.44 ± 2.87†‡§ (SE = 0.72)	14.13 ± 1.75*§ (SE = 0.44)	14.56 ± 1.67*§ (SE = 0.42)	16.38 ± 1.15*†‡ (SE = 0.29)

Note. RPE, rating of perceived exertion; AOP, arterial occlusion pressure; *p < 0.05 vs. 0% AOP; † p < 0.05 vs. 60% AOP; ‡ p < 0.05 vs. 70% AOP; § p < 0.05 vs. 80% AOP.

Table 7 shows the results of a one-way repeated measures ANOVA on RPE scores. Mauchly’s test indicated a violation of the sphericity assumption (p = 0.002); therefore, the Greenhouse–Geisser correction was applied (ε = 0.599). A significant main effect of occlusion pressure was observed: F (1.80, 26.94) = 25.34, p < 0.001, ηp^2^ = 0.63, 95% CI [0.28, 0.66], indicating that perceived exertion significantly increased with higher occlusion levels.

**TABLE 7:** Results of repeated measures ANOVA for RPE at different pressure levels.

Effect	F (df_1_, df_2_)	p-value	Partial η^2^	95% CI for partial η^2^
Pressure	25.34 (1.80, 26.94)	<0.001	0.63	[0.28, 0.66]

**FIGURE 4:**
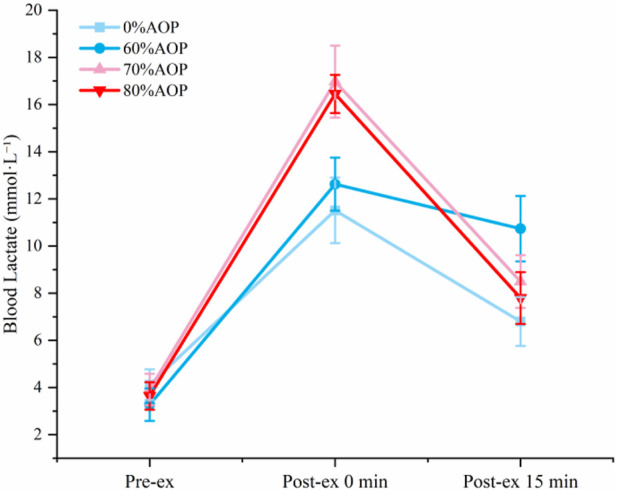
Changes in blood lactate concentration at different pressure levels.

Figure 4 illustrates changes in blood lactate concentrations, revealing that lactate levels immediately post-exercise at 70% and 80% AOP were significantly higher compared to 60% and 0% AOP (p < 0.05). Higher occlusion pressures likely produce greater local hypoxia, driving enhanced anaerobic metabolism and lactate production. This underscores that exercising under 70%–80% AOP elicits significantly greater metabolic stress than lower pressures. The equivalent lactate accumulation at 70% and 80% AOP (p > 0.05) implies that metabolic stress plateaus beyond 70% AOP.

**FIGURE 5:**
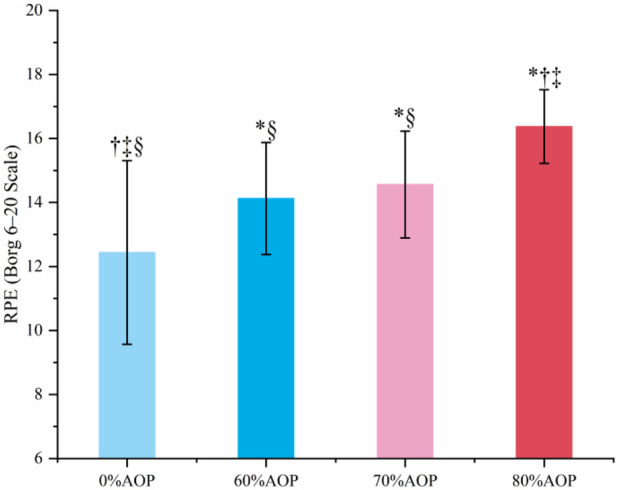
Post-hoc Test Results for the RPE Scores Under Different Pressure Levels (*p < 0.05 vs. 0% AOP; † p < 0.05 vs. 60% AOP; ‡ p < 0.05 vs. 70% AOP; § p < 0.05 vs. 80% AOP).

Figure 5 presents the post hoc test results of the RPE scores. Specifically, perceived exertion significantly increased with higher AOP levels (p < 0.05), with 80% AOP yielding the highest perceived exertion compared to lower pressures (p < 0.05). While 70% AOP resulted in significantly higher RPE than 0% AOP (p < 0.05), it remained significantly lower than that observed at 80% AOP (p < 0.05). Several subjects informally reported greater discomfort during the 80% AOP trial and requested that the cuff pressure be reduced mid-exercise. These comments were noted anecdotally during testing, but we did not administer a formal pain or discomfort scale (such as VAS Pain Scale) to quantify this feedback.

## 4 Discussion

Our findings clarify how acute physiological responses to low-load resistance exercise depend on the level of arterial occlusion. Both muscle activation (%MVC) and blood lactate were markedly elevated at 70% and 80% AOP compared to 60% AOP, whereas no significant differences emerged between 70% and 80%. In contrast, RPE was significantly higher at 80% than at 70% AOP, and several participants even requested cuff deflation at 80% due to discomfort (despite no formal pain scale being used). In short, pressures ≥70% AOP produced a strong physiological stimulus (higher muscle activation and lactate), but increasing pressure from 70% to 80% yielded no further increase in these markers while substantially increasing perceived effort.

In this study, the %MVC of lower limb muscles significantly increased through the BFRT protocol, which is consistent with previous findings (Fahs et al., 2015; Fatela et al., 2016; Loenneke et al., 2015a). This finding contributes to the existing body of knowledge by reinforcing the effectiveness of BFRT in enhancing muscle activation, which is crucial for muscle strength gains, and highlights the importance of individualized pressure settings to optimize training outcomes. From a metabolic perspective, BFRT induces a hypoxic environment within muscle tissue, leading to increased lactate accumulation and a concomitant decrease in pH levels (Kim et al., 2018; Pearson and Hussain, 2015). This shift toward anaerobic metabolism appears to be a central mechanism underlying the effects of BFRT. Hypoxia and decreased pH levels influence ATP rephosphorylation, stimulate the generation of reactive oxygen species (ROS) under hypoxic conditions, and alter cellular energy metabolism (Ferraro et al., 2014). As a result, hypoxia substantially impacts glycolysis and force production within muscle tissue (Horscroft and Murray, 2014). Furthermore, changes in myosin ATPase activity influence muscle recruitment patterns, resulting in varying degrees of muscle activation (Mijailovich et al., 2017). Notably, blood flow restriction affects capillary architecture and oxygen utilization, promoting muscle fiber adaptations and modulating motor neuron recruitment (Shimizu et al., 2016).

The enhanced muscle activation during BFRT can be attributed to metabolic “overload” within the muscle. During exercise, motor units are typically recruited according to the “size principle,” where smaller motor units are activated first, followed by larger ones. However, under conditions of restricted blood flow, the depletion of phosphocreatine stores and the decrease in intramuscular pH (Suga et al., 2010), coupled with insufficient oxygen supply to type I muscle fibers, lead to increased activation of type II muscle fibers, even at low intensities (Davies et al., 2016; Pearson and Hussain, 2015). Additionally, the reduction in oxygen supply and accumulation of metabolic byproducts due to restricted blood flow stimulate Type III and IV afferent nerves, further enhancing the recruitment of Type II fibers. This compensatory recruitment helps maintain muscle force and prevents motor neuron conduction failure during exercise (Wernbom and Aagaard, 2020).

In terms of muscle protein synthesis, key signaling proteins such as S6K1 are expressed at significantly higher levels in fast-twitch fibers compared to slow-twitch fibers (Qaisar et al., 2016; Tang et al., 2019). Low-intensity BFR training provides a novel mechanistic explanation for the activation of fast-twitch fibers, which were previously thought to be achievable only through intense physical stimuli, ultimately leading to the activation of muscle protein synthesis pathways. Moreover, lactate accumulation itself is increasingly recognized as a key metabolic signal for anabolic processes. Studies have shown that elevated lactate can stimulate the release of growth hormone and activate mTORC1 signaling pathways, which are essential for muscle protein synthesis (Deng et al., 2025; Kyun et al., 2024; Sumide et al., 2009). In this study, both 70% and 80% AOP produced significantly higher lactate concentrations than 60% AOP, suggesting that these levels may provide a sufficient metabolic stimulus for muscle adaptation. Mechanistically, lactate accumulation has long been proposed to enhance GH secretion and motor unit recruitment (Lim et al., 2022), and the current findings imply that only when occlusion is sufficient to build up lactate does GH/IGF-1 rise significantly (Dankel et al., 2017; Nalbandian and Takeda, 2016). Conversely, the lack of significant lactate response at 60% AOP may reflect insufficient metabolic stress, potentially limiting its efficacy in stimulating hypertrophic signaling. One explanation could be that partial venous occlusion at 60% AOP allowed sufficient oxygen delivery during low-load squatting, thereby blunting glycolytic flux and lactate production (Suga et al., 2010). Additionally, the lack of localized hypoxia may have reduced activation of fast-twitch muscle fibers, which are more reliant on anaerobic metabolism and therefore major contributors to lactate generation (Wernbom and Aagaard, 2020). These findings suggest that 60% AOP may fall below the “effective dose threshold” for BFR-induced metabolic signaling.

Notably, our results showed no significant differences in %MVC or lactate between 70% and 80% AOP, despite both being significantly higher than at 60% AOP. This pattern may indicate a physiological plateau effect, wherein pressures beyond 70% do not yield additional benefit in terms of muscle activation or metabolic stress. However, it is important to emphasize that this interpretation is speculative. The current findings only exhibit a plateau-like trend in the data; we do not claim to have definitively identified a plateau effect. More rigorous, targeted studies are needed to confirm whether such a threshold truly exists.

To further characterize the observed phenomenon, we conducted trend analyses examining the relationship between AOP level and acute physiological responses. The trend analysis for blood lactate concentration revealed a significant quadratic component across AOP levels, F (1, 45) = 4.22, p = 0.046, while the cubic component was not significant. This finding suggests that the dose–response relationship is not strictly linear, and instead may follow a curvilinear trajectory. Although we did not formally fit nonlinear regression models in the present study, the observed pattern implies that future research may benefit from modeling the pressure–response curve using polynomial or spline-based approaches to better define the optimal occlusion threshold. The observed lack of further increase in neuromuscular activation at pressures exceeding 70% AOP may be attributable to three interrelated physiological mechanisms: (1) Excessive compression at 80% AOP likely intensifies stimulation of group III/IV afferents by metabolites (H^+^, lactate, bradykinin) and mechanical stress. These nociceptive signals can reflexively inhibit ɑ-motoneuron output through spinal and supraspinal pathways, counteracting the drive for further muscle recruitment (Amann et al., 2013; Hug et al., 2015; Kennedy et al., 2015). Our observed RPE surge at 80% AOP supports this inhibitory feedback mechanism. (2) Near-complete arterial inflow restriction at ≥80% AOP critically compromises oxygen delivery and ATP resynthesis. While moderate hypoxia (70% AOP) potentiates type II fiber recruitment, severe hypoxia (80% AOP) may exceed the threshold where metabolic crisis impairs excitation-contraction coupling and force production (Gorgey et al., 2016; Scott et al., 2016). This aligns with Patterson et al.'s finding where pressures beyond “optimal occlusion” reduce metabolic stimulus efficiency (Patterson et al., 2019). (3) Cuff pressures approaching AOP (80%) may physically compress underlying neural structures and muscle spindles, potentially altering proprioceptive feedback and voluntary activation capacity (Wernbom et al., 2008). The discomfort reported by participants at 80% AOP likely reflects this mechanical stress.

The muscle cell swelling effect induced by BFRT is another key factor contributing to muscle hypertrophy (Loenneke et al., 2012). Muscle cell swelling increases intracellular pressure, which stimulates anabolic signaling pathways and promotes protein synthesis, thereby facilitating muscle growth. However, muscle cell swelling appears to reach a plateau beyond a certain level of blood flow restriction, indicating that further increases in pressure may not yield additional hypertrophic benefits. Indeed, elevated pressure levels exceeding 70% AOP may impair oxygen and nutrient delivery, leading to early muscle fatigue and an increased risk of injury (Ilett et al., 2019; Wernbom and Aagaard, 2020), which increases the risk of acute muscle fatigue and damage at 80% AOP, further suppressing muscle activation. Increased AOP levels may lead to reduced muscle oxygenation and accumulation of metabolic byproducts, ultimately worsening muscle fatigue and pain (Reis et al., 2019). Moreover, elevated pressure levels may activate sensory receptors in the nervous system, increasing perceived exertion and discomfort (Cockfield et al., 2024), potentially affecting exercise performance and adherence (Loenneke et al., 2015b; Mok et al., 2020). In the context of our study, the significantly elevated RPE observed at 80% AOP indicates a substantial increase in perceived effort and discomfort. Increasing the occlusion pressure beyond 70% AOP appears to yield diminishing physiological benefits while markedly increasing perceptual burden.

In this study, several participants reported excessive discomfort at 80% AOP, which hindered their ability to complete the required repetitions and led to requests for pressure reduction. However, it should be noted that RPE primarily reflects global exertion, not specific pain or discomfort. We did not employ a separate visual analog or pain scale for cuff discomfort, nor did we record any dedicated questionnaire on pressure-related pain. Our statement about greater discomfort at 80% was based on informal observation and participant comments.

While the present study provides valuable insight into the acute physiological responses to BFRT under varying occlusion pressures, several limitations must be acknowledged. First, all participants were healthy young males recruited from a single university cohort, which may limit the generalizability of the findings to females, older adults, clinical populations, or elite athletes. This gender homogeneity and potential volunteer bias should be addressed in future research through broader sampling strategies. Second, although individualized arterial occlusion pressures (AOP) were used, inter-individual variability in thigh circumference and tissue composition may influence actual occlusion levels, leading to discrepancies in physiological responses. Measuring AOP at rest may not fully reflect real-time occlusive effects during dynamic exercise due to acute changes in vascular tone and intramuscular pressure. Lastly, the modest sample size (n = 16) and non-randomized order of conditions may have introduced bias or limited statistical power to detect subtle effects, despite adequate power for primary outcomes.

Given these considerations, the findings of this study suggest that 70% AOP may provide a favorable balance between physiological efficacy and perceptual tolerability during low-load resistance training. However, caution is warranted in applying these pressures outside of athletic contexts. For clinical populations or novice trainees, higher AOP levels may pose discomfort or adherence challenges, and the safety profile of such protocols remains to be thoroughly assessed. Practitioners should therefore consider tailoring AOP intensity based on individual tolerance, training goals, and health status. Clear differentiation between athletic enhancement and clinical rehabilitation contexts is essential when prescribing BFRT.

Building on these implications, future research should pursue longitudinal intervention studies to evaluate the chronic adaptations to varying AOP levels, ideally employing mixed-effects modeling to account for inter- and intra-individual variability. Expanding the sample size (n > 20) and including diverse populations (e.g., females, older adults) would enhance external validity. Furthermore, integrating near-infrared spectroscopy (NIRS) could offer real-time insights into muscle oxygenation dynamics during BFRT, improving the physiological precision of occlusion monitoring. Such methodological enhancements will help refine BFRT protocols and identify optimal dosing strategies across populations and training conditions.

## 5 Conclusion

In conclusion, we propose that 70% AOP represents a potentially prudent trade-off in acute BFR exercise. It appears to elicit comparable metabolic and neuromuscular responses as 80% AOP, but with lower RPE and fewer subjective tolerance issues. However, this interpretation is tempered by the limitations of our discomfort assessment: we did not use a validated pain scale, and thus our observations of tolerability are qualitative. Future studies should include objective pain/discomfort measures (e.g., VAS pain scale) to confirm the relative tolerability of different pressures. For now, the evidence points toward 70% AOP as a practical choice that maximizes physiological stimulus while minimizing unnecessary discomfort. To strengthen these findings, future research should incorporate longitudinal designs with extended follow-up to evaluate chronic training adaptations, and employ mixed-effects modeling to account for inter-individual variability in pressure responses.

## Data Availability

The original contributions presented in the study are included in the article/supplementary material, further inquiries can be directed to the corresponding author.

## References

[B1] Abbuhl R. Gass S. Mackey A. (2013). Experimental research design. Res. methods linguistics 5 (1), 118–121. 10.1017/cbo9781139013734

[B2] Abe T. Sakamaki M. Fujita S. Ozaki H. Sugaya M. Sato Y. (2010). Effects of low-intensity walk training with restricted leg blood flow on muscle strength and aerobic capacity in older adults. Journal Geriatric Phys. Ther. 33 (1), 34–40. 10.1097/JPT.0b013e3181d07a73 20503732

[B3] Alwin D. F. (2007). Margins of error: a study of reliability in survey measurement. John Wiley and Sons 1 (2), 5–11. 10.1002/9780470146316

[B4] Amann M. Venturelli M. Ives S. J. McDaniel J. Layec G. Rossman M. J. (2013). Peripheral fatigue limits endurance exercise *via* a sensory feedback-mediated reduction in spinal motoneuronal output. J. Appl. physiology 115 (3), 355–364. 10.1152/japplphysiol.00049.2013 23722705 PMC3743006

[B5] Bond C. W. Hackney K. J. Brown S. L. Noonan B. C. (2019). Blood flow restriction resistance exercise as a rehabilitation modality following orthopaedic surgery: a review of venous thromboembolism risk. J. Orthop. and Sports Phys. Ther. 49 (1), 17–27. 10.2519/jospt.2019.8375 30208794

[B6] Brooks J. L. (2012). Counterbalancing for serial order carryover effects in experimental condition orders. Psychol. methods 17 (4), 600–614. 10.1037/a0029310 22799624

[B7] Clarkson M. J. May A. K. Warmington S. A. (2020). Is there rationale for the cuff pressures prescribed for blood flow restriction exercise? A systematic review. Scand. J. Med. and Sci. sports 30 (8), 1318–1336. 10.1111/sms.13676 32279391

[B8] Cockfield B. A. Wedig I. J. Vinckier A. L. McDaniel J. Elmer S. J. (2024). Physiological and perceptual responses to acute arm cranking with blood flow restriction. Eur. J. Appl. physiology 124 (5), 1509–1521. 10.1007/s00421-023-05384-0 38142449

[B9] Cook C. J. Kilduff L. P. Beaven C. M. (2014). Improving strength and power in trained athletes with 3 weeks of occlusion training. Int. J. sports physiology Perform. 9 (1), 166–172. 10.1123/ijspp.2013-0018 23628627

[B10] Dankel S. J. Mattocks K. T. Jessee M. B. Buckner S. L. Mouser J. G. Loenneke J. P. (2017). Do metabolites that are produced during resistance exercise enhance muscle hypertrophy? Eur. J. Appl. physiology 117, 2125–2135. 10.1007/s00421-017-3690-1 28776271

[B11] Das A. Paton B. (2022). Is there a minimum effective dose for vascular occlusion during blood flow restriction training? Front. physiology 13, 838115. 10.3389/fphys.2022.838115 35464074 PMC9024204

[B12] Davies T. Orr R. Halaki M. Hackett D. (2016). Effect of training leading to repetition failure on muscular strength: a systematic review and meta-analysis. Sports Med. 46 (1), 487–502. 10.1007/s40279-015-0451-3 26666744

[B63] Deng B. Lin G. Shi Y. Li D. Guan Z. Liang C. (2025). The effects of blood flow restriction combined with resistance training on lower limb strength, muscle hypertrophy, jumping ability, and sprint speed in athletes: a systematic review and meta-analysis. Front. Physiol. 16, 1612685. 10.3389/fphys.2025.1612685 40800729 PMC12339475

[B13] de Queiros V. S. Rolnick N. Wilde P. de Melo A. Cabral B. G. Dantas P. M. (2024). Measuring arterial occlusion pressure for training with blood flow restriction: a scoping review and recommendations for measurement. Sport Sci. Health 20 (2), 259–272. 10.1007/s11332-023-01135-y

[B14] Fahs C. A. Loenneke J. P. Thiebaud R. S. Rossow L. M. Kim D. Abe T. (2015). Muscular adaptations to fatiguing exercise with and without blood flow restriction. Clin. physiology Funct. imaging 35 (3), 167–176. 10.1111/cpf.12141 24612120

[B15] Fatela P. Reis J. F. Mendonca G. V. Avela J. Mil-Homens P. (2016). Acute effects of exercise under different levels of blood-flow restriction on muscle activation and fatigue. Eur. J. Appl. physiology 116, 985–995. 10.1007/s00421-016-3359-1 27017495

[B16] Ferraro E. Giammarioli A. M. Chiandotto S. Spoletini I. Rosano G. (2014). Exercise-induced skeletal muscle remodeling and metabolic adaptation: redox signaling and role of autophagy. Antioxidants and redox Signal. 21 (1), 154–176. 10.1089/ars.2013.5773 24450966 PMC4048572

[B17] Frost J. (2015). Repeated measures designs: benefits, challenges and an ANOVA example. Minitab Blog.

[B18] Geng Y. U. Zhang L. Wu X. (2021). Effects of blood flow restriction training on blood perfusion and work ability of muscles in elite para-alpine skiers. Med. Sci. Sports Exerc. 54 (3), 489–496. 10.1249/MSS.0000000000002805 34669671 PMC8830888

[B19] Gorgey A. S. Timmons M. K. Dolbow D. R. Bengel J. Fugate-Laus K. C. Michener L. A. (2016). Electrical stimulation and blood flow restriction increase wrist extensor cross-sectional area and flow meditated dilatation following spinal cord injury. Eur. J. Appl. physiology 116, 1231–1244. 10.1007/s00421-016-3385-z 27155846

[B20] Greenhouse S. W. Geisser S. (1959). On methods in the analysis of profile data. Psychometrika 24 (2), 95–112. 10.1007/bf02289823

[B21] Hackney K. J. Brown L. W. J. Stone K. A. Tennent D. J. (2018). The role of blood flow restriction training to mitigate sarcopenia, dynapenia, and enhance clinical recovery. Tech. Orthop. 33 (2), 98–105. 10.1097/bto.0000000000000271

[B22] Hart H. C. Blazzard C. Kasper N. Lacey R. Lopez D. Richards S. (2022). Differences in arterial occlusion pressure as measured using ultrasound and a hand-held doppler device. Int. J. Exerc. Sci. Conf. Proc. 14 (2), 131.

[B23] Hermens H. J. Freriks B. Disselhorst-Klug C. Rau G. (2000). Development of recommendations for SEMG sensors and sensor placement procedures. J. Electromyogr. Kinesiol. 10 (5), 361–374. 10.1016/s1050-6411(00)00027-4 11018445

[B24] Horscroft J. A. Murray A. J. (2014). Skeletal muscle energy metabolism in environmental hypoxia: climbing toward consensus. Extreme physiology and Med. 3 (1), 1–17. 10.1186/2046-7648-3-19 PMC4253994 25473486

[B25] Hug F. Tucker K. Gennisson J. L. Tanter M. Nordez A. (2015). Elastography for muscle biomechanics: toward the estimation of individual muscle force. Exerc. sport Sci. Rev. 43 (3), 125–133. 10.1249/JES.0000000000000049 25906424

[B64] Hughes L. Paton B. Rosenblatt B. Gissane C. Patterson S. D. (2017). Blood flow restriction training in clinical musculoskeletal rehabilitation: a systematic review and meta-analysis. Br J Sports Med. 51 (13), 1003–1011. 10.1136/bjsports-2016-097071 28259850

[B26] Hunt J. E. Stodart C. Ferguson R. A. (2016). The influence of participant characteristics on the relationship between cuff pressure and level of blood flow restriction. Eur. J. Appl. physiology 116 (1), 1421–1432. 10.1007/s00421-016-3399-6 27235157 PMC4911379

[B27] Ilett M. J. Rantalainen T. Keske M. A. May A. K. Warmington S. A. (2019). The effects of restriction pressures on the acute responses to blood flow restriction exercise. Front. physiology 10 (1), 1018. 10.3389/fphys.2019.01018 31456694 PMC6700307

[B28] Jessee M. B. Buckner S. L. Dankel S. J. Counts B. R. Abe T. Loenneke J. P. (2016). The influence of cuff width, sex, and race on arterial occlusion: implications for blood flow restriction research. Sports Med. 46 (1), 913–921. 10.1007/s40279-016-0473-5 26820301

[B29] Jessee M. B. Mattocks K. T. Buckner S. L. Dankel S. J. Mouser J. G. Abe T. (2018). Mechanisms of blood flow restriction: the new testament. Tech. Orthop. 33 (2), 72–79. 10.1097/bto.0000000000000252

[B30] Kennedy D. S. Fitzpatrick S. C. Gandevia S. C. Taylor J. L. (2015). Fatigue-related firing of muscle nociceptors reduces voluntary activation of ipsilateral but not contralateral lower limb muscles. J. Appl. physiology 118 (4), 408–418. 10.1152/japplphysiol.00375.2014 25525208

[B31] Kim T. H. Lee S. H. Kim Y. J. Kim S. J. Kang J. H. Kwak H. B. (2018). Effect of acute resistance exercise with different level of blood flow restriction on acute changes in muscle thickness, blood lactate, CK, and oxidative stress in Male adults. Exerc. Sci. 27 (1), 50–61. 10.15857/ksep.2018.27.1.50

[B32] Konrad P. (2005). The abc of emg. A practical introduction to kinesiological electromyography, 1 (1), 30–51.

[B65] Kyun S. Kim J. Hwang D. Jang I. Park H. Y. Lim K. (2024). Lactate administration induces skeletal muscle synthesis by influencing Akt/mTOR and MuRF1 in non-trained mice but not in trained mice. Physiol Rep. 12 (4), e15952. 10.14814/phy2.15952 38383135 PMC10881281

[B33] Lim C. Nunes E. A. Currier B. S. Mcleod J. C. Thomas A. C. Phillips S. M. (2022). An evidence-based narrative review of mechanisms of resistance exercise–induced human skeletal muscle hypertrophy. Med. Sci. sports Exerc. 54 (9), 1546–1559. 10.1249/MSS.0000000000002929 35389932 PMC9390238

[B34] Loenneke J. Fahs C. A. Rossow L. M. Abe T. Bemben M. G. (2012). The anabolic benefits of venous blood flow restriction training may be induced by muscle cell swelling. Med. hypotheses 78 (1), 151–154. 10.1016/j.mehy.2011.10.014 22051111

[B35] Loenneke J. Thiebaud R. S. Fahs C. A. Rossow L. M. Abe T. Bemben M. G. (2014). Blood flow restriction: effects of cuff type on fatigue and perceptual responses to resistance exercise. Acta Physiol. Hung. 101 (2), 158–166. 10.1556/APhysiol.101.2014.2.4 24901077

[B36] Loenneke J. P. Kim D. Fahs C. A. Thiebaud R. S. Abe T. Larson R. D. (2015a). Effects of exercise with and without different degrees of blood flow restriction on torque and muscle activation. Muscle and nerve 51 (5), 713–721. 10.1002/mus.24448 25187395

[B37] Loenneke J. P. Kim D. Fahs C. A. Thiebaud R. S. Abe T. Larson R. D. (2015b). The effects of resistance exercise with and without different degrees of blood-flow restriction on perceptual responses. J. sports Sci. 33 (14), 1472–1479. 10.1080/02640414.2014.992036 25555163

[B67] Masri B. A. Day B. Younger A. S. Jeyasurya J. (2016). Technique for measuring limb occlusion pressure that facilitates personalized tourniquet systems: a randomized trial J. Med. Biol. Eng. 36 (5), 644–650. 10.1007/s40846-016-0173-5 27853415 PMC5083760

[B38] McEwen J. A. Owens J. G. Jeyasurya J. (2019). Why is it crucial to use personalized occlusion pressures in blood flow restriction (BFR) rehabilitation? J. Med. Biol. Eng. 39, 173–177. 10.1007/s40846-018-0397-7

[B39] Mijailovich S. M. Nedi c D. Svicevi c M. Stojanovic B. Walklate J. Ujfalusi Z. (2017). Modeling the actin. myosin ATPase cross-bridge cycle for skeletal and cardiac muscle myosin isoforms. Biophysical J. 112 (5), 984–996. 10.1016/j.bpj.2017.01.021 PMC5355499 28297657

[B40] Mok E. Suga T. Sugimoto T. Tomoo K. Dora K. Takada S. (2020). Negative effects of blood flow restriction on perceptual responses to walking in healthy young adults: a pilot study. Heliyon 6 (8), e04745–515. 10.1016/j.heliyon.2020.e04745 32885079 PMC7452548

[B41] Mouser J. G. Dankel S. J. Jessee M. B. Mattocks K. T. Buckner S. L. Counts B. R. (2017). A tale of three cuffs: the hemodynamics of blood flow restriction. Eur. J. Appl. physiology 117, 1493–1499. 10.1007/s00421-017-3644-7 28501908

[B42] Nalbandian M. Takeda M. (2016). Lactate as a signaling molecule that regulates exercise-induced adaptations. Biology 5 (4), 38. 10.3390/biology5040038 27740597 PMC5192418

[B43] Nascimento D. D. C. Rolnick N. Neto I. V. D. S. Severin R. Beal F. L. R. (2022). A useful blood flow restriction training risk stratification for exercise and rehabilitation. Front. physiology 318. 10.3389/fphys.2022.808622 35360229 PMC8963452

[B44] Patterson S. D. Hughes L. Warmington S. Burr J. Scott B. R. Owens J. (2019). Blood flow restriction exercise: considerations of methodology, application, and safety. Front. physiology 2 (3), 533. 10.3389/fphys.2019.00533 31156448 PMC6530612

[B45] Pearson S. J. Hussain S. R. (2015). A review on the mechanisms of blood-flow restriction resistance training-induced muscle hypertrophy. Sports Med. 45 (1), 187–200. 10.1007/s40279-014-0264-9 25249278

[B46] Pignanelli C. Christiansen D. Burr J. F. (2021). Blood flow restriction training and the high-performance athlete: science to application. J. Appl. Physiology 1 (1), 23–33. 10.1152/japplphysiol.00982.2020 33600282

[B47] Qaisar R. Bhaskaran S. Van Remmen H. (2016). Muscle fiber type diversification during exercise and regeneration. Free Radic. Biol. Med. 98 (1), 56–67. 10.1016/j.freeradbiomed.2016.03.025 27032709

[B48] Reis J. F. Fatela P. Mendonca G. V. Vaz J. R. Valamatos M. J. Infante J. (2019). Tissue oxygenation in response to different relative levels of blood-flow restricted exercise. Front. physiology 10, 407. 10.3389/fphys.2019.00407 31031637 PMC6470188

[B49] Roehl T. Lambert B. S. Ankersen J. Hernandez K. McCulloch P. C. Hedt C. (2023). Optimal blood flow restriction occlusion pressure for shoulder muscle recruitment with upper extremity exercise. Am. J. Sports Med. 51 (7), 1859–1871. 10.1177/03635465231166959 37092707

[B50] Scott B. R. Loenneke J. P. Slattery K. M. Dascombe B. J. (2016). Blood flow restricted exercise for athletes: a review of available evidence. J. Sci. Med. sport 19 (5), 360–367. 10.1016/j.jsams.2015.04.014 26118847

[B51] Segal N. A. Williams G. N. Davi s M. C. Wallace R. B. Mikesky A. E. (2015). Efficacy of blood flow–restricted, low-load resistance training in women with risk factors for symptomatic knee osteoarthritis. Pm&r 7 (4), 376–384. 10.1016/j.pmrj.2014.09.014 25289840 PMC4385750

[B52] Shimizu R. Hotta K. Yamamoto S. Matsumoto T. Kamiya K. Kato M. (2016). Low-intensity resistance training with blood flow restriction improves vascular endothelial function and peripheral blood circulation in healthy elderly people. Eur. J. Appl. physiology 116, 749–757. 10.1007/s00421-016-3328-8 26822582

[B53] Spranger M. D. Krishnan A. C. Levy P. D. O'Leary D. S. Smith S. A. (2015). Blood flow restriction training and the exercise pressorreflex: a call for concern. Am. J. Physiology-Heart Circulatory Physiology 309 (9), 1440–1452. 10.1152/ajpheart.00208.2015 26342064 PMC7002872

[B54] Suga T. Okita K. Morita N. Yokota T. Hirabayashi K. Horiuchi M. (2010). Dose effect on intramuscular metabolic stress during low-intensity resistance exercise with blood flow restriction. J. Appl. physiology 108 (6), 1563–1567. 10.1152/japplphysiol.00504.2009 20360434

[B66] Sumide T. Sakuraba K. Sawaki K. Ohmura H. Tamura Y. (2009). Effect of resistance exercise training combined with relatively low vascular occlusion. J. Sci. Med. Sport. 12 (1), 107–112. 10.1016/j.jsams.2007.09.009 18083635

[B55] Tabata S. Suzuki Y. Azuma K. Matsumoto H. (2016). Rhabdomyolysis after performing blood flow restriction training: a case report. J. strength Cond. Res. 30 (7), 2064–2068. 10.1519/JSC.0000000000001295 26677831

[B56] Tang H. Inoki K. Brooks S. V. Okazawa H. Lee M. Wang J. (2019). mTORC1 underlies age‐related muscle fiber damage and loss by inducing oxidative stress and catabolism. Aging cell 18 (3), e12943. 10.1111/acel.12943 30924297 PMC6516169

[B57] Vasey M. W. Thayer J. F. (1987). The continuing problem of false positives in repeated measures ANOVA in psychophysiology: a multivariate solution. Psychophysiology 24 (4), 479–486. 10.1111/j.1469-8986.1987.tb00324.x 3615759

[B58] Wernbom M. Aagaard P. (2020). Muscle fiber activation and fatigue with low‐load blood flow restricted resistance exercise: an integrative physiology review. Acta physiol. 228 (1), 13–32. 10.1111/apha.13302 31108025

[B59] Wernbom M. Augustsson J. Raastad T. (2008). Ischemic strength training: a low‐load alternative to heavy resistance exercise? Scand. J. Med. and Sci. sports 18 (4), 401–416. 10.1111/j.1600-0838.2008.00788.x 18466185

[B60] Yasuda T. Fujita T. Miyagi Y. Kubota Y. Sato Y. Nakajima T. (2006). Electromyographic responses of arm and chest muscle during bench press exercise with and without KAATSU. Int. J. KAATSU Train. Res. 2 (1), 15–18. 10.3806/ijktr.2.15

[B61] Yasuda T. Fukumura K. Fukuda T. Iida H. Imuta H. Sato Y. (2014). Effects of low‐intensity, elastic band resistance exercise combined with blood flow restriction on muscle activation. Scand. J. Med. and Sci. sports 24 (1), 55–61. 10.1111/j.1600-0838.2012.01489.x 22734915

[B62] Yuan J. Wu L. Xue Z. Xu G. Wu Y. (2023). Application and progress of blood flow restriction training in improving muscle mass and strength in the elderly. Front. physiology 14, 1155314. 10.3389/fphys.2023.1155314 37035674 PMC10079911

